# Distance to Specialist Medical Care and Diagnosis of Obstructive Sleep Apnea in Rural Saskatchewan

**DOI:** 10.1155/2019/1683124

**Published:** 2019-01-14

**Authors:** Catherine M. Spagnuolo, Michael McIsaac, James Dosman, Chandima Karunanayake, Punam Pahwa, William Pickett

**Affiliations:** ^1^Department of Public Health Sciences, Queen's University, Kingston, Ontario K7L 3N6, Canada; ^2^Canadian Centre for Health and Safety in Agriculture, University of Saskatchewan, Saskatoon, Saskatchewan S7N 2Z4, Canada

## Abstract

Obstructive sleep apnea (OSA) is the most common sleep-disordered breathing condition. Patients with OSA symptoms are often not diagnosed clinically, which is a concern, given the health and safety risks associated with unmanaged OSA. The availability of fewer practicing medical specialists combined with longer travel distances to access health care services results in barriers to diagnosis and treatment in rural communities. This study aimed to (1) determine whether the proportion of adults reporting OSA symptoms in the absence of a sleep apnea diagnosis in rural populations varied by travel distance to specialist medical care and (2) assess whether any distance-related patterns were attributable to differences in the frequency of diagnosis among adults who likely required this specialist medical care. We used a cross-sectional epidemiologic study design, augmented by analysis of follow-up survey data. Our study base included adults who completed a 2010 baseline questionnaire for the Saskatchewan Rural Health Study. Follow-up occurred until 2015. 6525 adults from 3731 households constituted our sample. Statistical models used log-binomial regression. Rural adults who reported the largest travel distances (≥250 km) to specialist medical care were 1.17 (95% CI: 1.07, 1.29) times more likely to report OSA symptoms in the absence of a sleep apnea diagnosis than those who reported the smallest (<100 km; referent) distances. However, the proportion of sleep apnea diagnoses was low and unaffected by reported travel distance among adults who likely required this specialist medical care. Our findings suggest factors other than travel distance may be contributing to the low sleep apnea diagnostic rate. This remains important as undiagnosed and untreated OSA has serious implications on the health of people and populations, but effective treatments are available. Health care access barriers to the diagnosis and treatment of OSA require evaluation to inform health care planning and delivery.

## 1. Introduction

Obstructive sleep apnea (OSA) is the most common sleep-disordered breathing condition [1, 2]. It is estimated that 5.4 million Canadian adults have symptoms of OSA, yet most symptomatic adults are not clinically diagnosed [1–4]. OSA is characterized by periodic obstructions of the upper airway during sleep, leading to complete cessation (apnea) or reduction (hypopnea) in airflow [5–7]. Sleep-disturbed breathing may result in reduction of hemoglobin oxygen concentration (hypoxemia) and fragmented sleep [5, 8]. Patient reports of excessive daytime sleepiness and loud snoring are common diagnostic symptoms [7, 9]. If untreated, symptoms may interfere with activities of daily living and increase risks for occupational injury [1, 10–12], motor vehicle crashes [13, 14], and cardiovascular diseases [15–24]. Effective treatments for OSA exist [25–37] and can substantially improve health outcomes and overall quality of life. The most common treatment, continuous positive airway pressure (CPAP), assists in mitigating health and safety risks [14, 23, 38–44].

Rural populations may be particularly at risk for OSA due to high levels of obesity, physical inactivity, and other related risk factors [45–50]. Rural patients with OSA symptoms are often not diagnosed clinically [1], which is a concern, as the increased general health risks experienced by rural vs. urban residents [46, 51–57] may be exacerbated specifically by the presence of undiagnosed OSA. Explanations for such clinical patterns warrant focused study.

Access to health care services is an important determinant of health [58, 59], and health care use is known to be lower in rural than in urban communities [60–63]. The availability of fewer practicing medical specialists combined with longer travel distances to access health care services results in barriers to diagnosis and treatment [47, 55, 56, 64–67]. Care for patients with clinical suspicion of OSA begins with the family physician and, if necessary, the family doctor then refers the patient for diagnostic testing at a sleep clinic [68]. At the specialist level, long travel distance may be an important barrier to the diagnosis and treatment of sleep apnea in rural populations, given modest numbers of sleep laboratories [3, 69]. Travel distance is an example of the availability and accommodation dimension of health care access (as per Levesque's model [70]). Even with access to a family doctor, travel distance to specialist care is still a relevant concern for sleep apnea because referral to a sleep medicine specialist for overnight, in-laboratory diagnostic testing is the gold standard for diagnosis [71, 72].

Lack of diagnosis and treatment for OSA is concerning, and evidence-informed changes to health service planning in rural communities surrounding the diagnosis and treatment of OSA may be particularly impactful. While a number of studies have evaluated the impact of rural residence and/or travel burden on the use of specialized health care services (e.g., mental health services, screening for colorectal, and breast and cervical cancer) and clinical diagnoses such as cancer [60–63, 66, 67, 73, 74], to our knowledge, no Canadian studies have specifically examined the impacts of travel distance to specialist medical services on sleep apnea diagnosis. We had the opportunity to address this gap in knowledge using baseline and follow-up data from a large population-based rural health study conducted in Saskatchewan from 2010 to 2015 [75]. Our hope was to contribute to policy decisions whereby the diagnosis and effective treatment of rural people with sleep apnea would be improved.

## 2. Materials and Methods

### 2.1. Data Source: The Saskatchewan Rural Health Study

The Saskatchewan Rural Health Study is a population-based cohort study. The baseline phase occurred in 2010 and the follow-up phase began in 2014 and ended in 2015. Both phases involved administration of a survey developed based on the population health framework [75, 76] that documented individual and contextual factors that characterized and influenced respiratory health, including sleep-disordered breathing [75].

#### 2.1.1. Baseline Sample

Rural municipalities and towns (population ranging from 500 to 5000 [75]) were selected from four quadrants of the province (northwest, northeast, southwest, and southeast). A random sample of 36 of the 297 rural municipalities in Saskatchewan (nine per quadrant) and 16 of Saskatchewan's 145 towns were selected to participate. Local councils for 32 of the rural municipalities and 15 of the towns agreed to participate on behalf of their residents and provided mailing addresses. Eligible towns and municipalities were those that (1) were located at least 60 km from an urban center, meeting the Statistics Canada definition of “rural” [75, 77]; (2) were located outside the commuting zone of larger urban centers (areas with a population of 10,000 or more [75, 77]); (3) did not recently participate in the Saskatchewan Farm Injury Cohort Study, another large cohort study [75, 78]; and (4) were not included in the pilot study conducted to refine the baseline questionnaire and inform methods to optimize response rates [75, 79]. A registry of mailing addresses compiled from taxation lists was used to determine household eligibility. Households with unknown or duplicate addresses were excluded, as were homeowners with a mailing address outside the study area and deceased homeowners. Eligible adults were all those aged 18 years and older living in included households.

#### 2.1.2. Baseline Data Collection

Data were collected by self-administered questionnaire using Dillman's total design method [80, 81] to optimize response rates. The household response rate was 42% [75]. A key informant was asked to provide household-level information and then to complete a section for each adult (aged 18 years and older) living in the household. Data were collected for 8261 adults from 4624 households in farm and nonfarm rural communities in Saskatchewan. Our study base included adult participants who completed the baseline questionnaire.

#### 2.1.3. Follow-Up Phase: Sample and Data Collection

The baseline sample was subsequently contacted for participation in the follow-up phase of the study [82]. Consenting adults comprised this sample. Follow-up data were collected for 4867 adults from 2797 households by a similar survey (household response rate = 60%; individual response rate = 59%) [82].

### 2.2. Study Design

**Figure 1:**
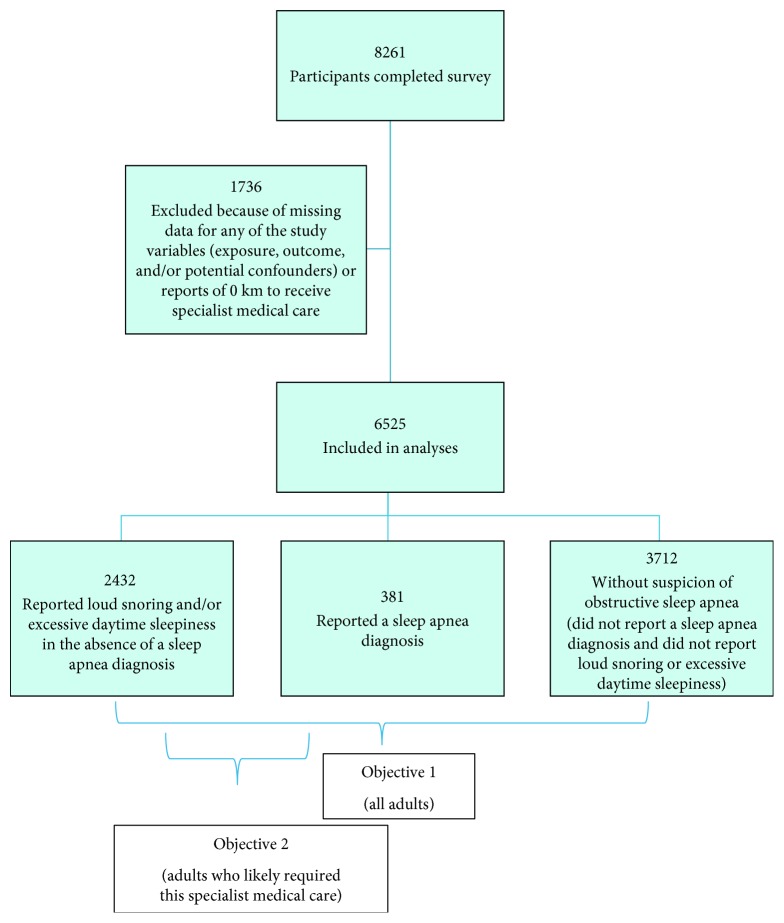
Sample according to self-reports of a sleep apnea diagnosis and obstructive sleep apnea symptoms (loud snoring and excessive daytime sleepiness measured by an Epworth Sleepiness Scale score of greater than 10 out of 24).

Cross-sectional analyses of baseline data were performed. Adults were classified according to reports of a sleep apnea diagnosis and the common OSA symptoms of loud snoring and excessive daytime sleepiness as assessed by the Epworth Sleepiness Scale (ESS) score [7, 9]. Out of the 8261 participants in the baseline sample, 6525 adults from 3731 households provided complete information for all variables of interest in our study (exposure, outcome, and/or potential confounders) and were included in analyses (Figure 1).

Analysis of follow-up survey data aimed to assess whether, after five years, the frequency of an incident sleep apnea diagnosis among adults reporting OSA symptoms in the absence of a sleep apnea diagnosis at baseline was attributable to travel distance to specialist medical care. Out of the 4867 participants in the follow-up phase sample, 1459 adults from 1180 households met the inclusion criteria for this analysis and provided complete information for all variables of interest in our study.

## 3. Measurement of Key Variables

### 3.1. Objective 1

#### 3.1.1. Exposure: Travel Distance to Specialist Medical Care

Travel distance to specialist medical care was assessed using an original survey item developed to evaluate access to health care services that asked: “How far do you travel to receive medical or surgical specialist services (in km)?” [75]. Distance quartiles were established: (1) <100 km, (2) 100–189 km, (3) 190–249 km, and (4) ≥250 km.

#### 3.1.2. Outcome

The outcome was self-reports of OSA symptoms (loud snoring and/or excessive daytime sleepiness) in the absence of a sleep apnea diagnosis and was assessed using the survey items for sleep apnea diagnosis and OSA symptoms.

#### 3.1.3. Sleep Apnea Diagnosis

This diagnosis was determined using the survey item adapted from the Canadian Community Health Survey that asked, “Has a doctor ever said you (yes or no) had any of the following chest illnesses (ever in your life and during the past 12 months): h. Sleep Apnea” [83].

#### 3.1.4. OSA Symptoms

A dichotomous “loud snoring” variable (yes or no) was created based on the following 2 original survey items: (1) “Do you snore?” and (2) “If you snore, is your snoring: Slightly louder than breathing? As loud as talking? Louder than talking? Very loud—can be heard in adjacent rooms?” [75]. Loud snoring was identified in adults who reported snoring that was “as loud as talking,” “louder than talking,” or “very loud” [84].

The ESS score was used to assess excessive daytime sleepiness [75]. The scale described eight situations and asked, “How likely are you to doze off or fall asleep in the situations described below, in contrast to just feeling tired? This refers to your usual way of life in recent times. Even if you have not done some of these things recently, try to work out how they would have affected you. Please check one response choice for each situation.” Likelihood of dozing off or falling asleep was scored on a 4-point (0–3) Likert scale. The eight responses were summed, and a score greater than 10 out of 24 was considered abnormal and indicative of excessive daytime sleepiness [7, 85, 86].

### 3.2. Objective 2

#### 3.2.1. Exposure: Travel Distance to Specialist Medical Care

Travel distance to specialist medical care was assessed in the same way as described for objective 1.

#### 3.2.2. Outcome

The outcome was reports of a sleep apnea diagnosis among adults who likely required this specialist medical care (e.g., adults reporting either OSA symptoms or a sleep apnea diagnosis), which was assessed using the survey item for sleep apnea diagnosis used for objective 1.

## 4. Minimum Detectable Relative Risks and Attributable Risk

The minimum detectable relative risk represents the smallest increase in risk for reporting the outcome between the farthest and closest distance quartiles that our study can detect with 80% power and an alpha level (two-sided) of 5%. We could detect a relative risk of 1.32 or greater for the first objective and 1.55 or greater for the second objective.

We calculated an attributable risk percent of 24%. This means that 24% of adults reporting the largest travel distances to specialist medical care (≥250 km) who also report OSA symptoms in the absence of a sleep apnea diagnosis could have potentially been diagnosed had their travel distances been shorter (e.g., improved access to specialist care). A priori, we considered that because of the health and safety risks associated with unmanaged OSA [1, 10–24, 38–44], combined with the availability of effective treatments [25–37], this makes this 24% increase in reports of OSA symptoms in the absence of a sleep apnea diagnosis clinically important. Providing these individuals with appropriate care may help manage symptoms and reduce health and safety risks at the individual and population levels [14, 23, 38–44].

### 4.1. Statistical Analyses

All analyses were conducted using SAS software version 9.4 (SAS Institute Inc., Cary, NC).

#### 4.1.1. Sample Description

Characteristics of the study sample, as well as the subset of adults who likely required this specialist medical care, were described by each study variable according to (1) the primary outcome, reports of OSA symptoms in the absence of a sleep apnea diagnosis, and (2) travel distance to specialist medical care. Next, proportions of individuals reporting OSA symptoms in the absence of a sleep apnea diagnosis, both overall and within strata of each study variable, were examined. Rao-Scott chi-square tests [87], which adjust for the clustered nature of the data (individuals nested within households), were performed to test for statistical significance of differences in proportions. The same was done to compare proportions of individuals in the farthest distance quartile, both overall and within strata of each study variable.

#### 4.1.2. Regression Analysis

Log-binomial regression models were created to estimate the strength of associations between (1) travel distance to specialist medical care and the proportion of adults reporting OSA symptoms in the absence of a sleep apnea diagnosis and (2) travel distance to specialist medical care and frequency of sleep apnea diagnosis among adults who likely required this specialist medical care. Sex was first explored as an effect modifier. Models then accounted for potential confounders (age, sex, “money left over at the end of the month,” and education level) as well as body mass index (BMI), heavy alcohol consumption, and smoking status, which were treated as proxies for baseline health behaviors (unmeasured potential confounders) to reduce any differences that might arise as reasons other than travel distance to specialist medical care. To account for clustering of adults within households, generalized estimating equations (exchangeable working correlation structure) were used to obtain robust variance estimates [88]. Relative risks and their 95% confidence intervals were estimated.

We also evaluated comparability of reported travel distance to specialist medical care and driving distance to the closest of Saskatchewan's sleep centers for all households using postal codes.

#### 4.1.3. Sensitivity Analysis

A sensitivity analysis using multiple imputation [89] was performed to assess the potential impact of excluding participants with missing data.

## 5. Results

### 5.1. Objective 1

Six percent (381/6525) of the sample reported a sleep apnea diagnosis and an additional 37% (2432/6525) reported OSA symptoms in the absence of a sleep apnea diagnosis (Figure 1). Participants ranged in age from 18 to 101 years (mean 55.0 [±15.6] years), with a mean reported travel distance to specialist medical care of 183 [±113] km (Table 1). The largest reported travel distances (≥250 km) were significantly and positively associated with lower education levels and “not enough” money left over at the end of the month (Table 1). There was an increased proportion of adults reporting OSA symptoms in the absence of a sleep apnea diagnosis in association with increasing travel distance to specialist medical care (Table 1). Middle to older age, male sex, overweight and obese BMIs, secondary education or less, higher frequency of heavy alcohol consumption, and a history of smoking were also associated with higher proportions of adults reporting OSA symptoms in the absence of a sleep apnea diagnosis (Table 1).

**Figure 2:**
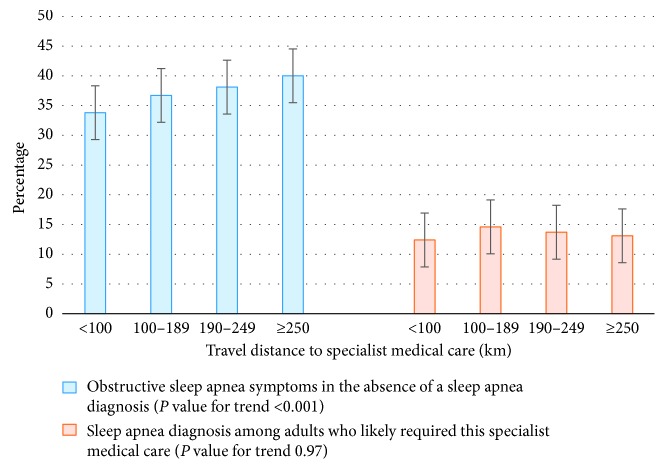
Proportion of adults reporting (1) obstructive sleep apnea symptoms in the absence of a sleep apnea diagnosis and (2) a sleep apnea diagnosis by travel distance to specialist medical care. The error bars represent the standard error of the mean.

Adults who reported the largest travel distances (≥250 km) to access specialist medical care were 19% more likely to report OSA symptoms in the absence of a sleep apnea diagnosis than those who reported the smallest travel distances (<100 km) (RR = 1.19; 95% CI: 1.08, 1.30; Table 2). The relative risks and 95% CIs were not statistically different between males and females at the 5% level of significance, after adjustment for confounding variables (*P*=0.06). Sex was not found to be a meaningful effect modifier and was controlled for as a potential confounder. After adjustment for confounding variables, the effect of travel distance to specialist medical care was similar to the unadjusted effect (RR = 1.17; 95% CI: 1.07, 1.29; Table 2). There was a significant increasing trend (*P* < 0.001) between the proportion of adults reporting OSA symptoms in the absence of a sleep apnea diagnosis and travel distance to specialist medical care (Figure 2).

Our comparison of reported travel distance to specialist medical care and driving distance to the closest of Saskatchewan's sleep centers for all households using postal codes found inconsistencies between reported travel distances and driving distance. For example, among the adults who reported distances between 100 km and 189 km to access specialist medical care, driving distance to the closest sleep center in the province was consistent with reported data only 36% of the time; driving distance was larger than reported distance 60% of the time. Among the adults who reported distances of 250 km or greater to access specialist medical care, driving distance to the closest sleep center in the province was smaller than reported distance 38% of the time (Table 3). Distance as determined by postal code may be more of an approximation than distances reported by rural dwellers whose way of life depends on extensive travel.

### 5.2. Objective 2

Forty-three percent (2813/6525) of the sample was identified as possibly requiring sleep specialist care (either reported OSA symptoms or a sleep apnea diagnosis) (Figure 1). This subset of the sample is described in Table 4. Age ranged from 18 to 101 (mean 55.5 [±13.6]) years. Mean travel distance to specialist medical care was 189 [±114] km; mean distance was similar among adults reporting a sleep apnea diagnosis and those reporting OSA symptoms in the absence of a sleep apnea diagnosis (188 [±113] km vs. 189 [±114] km, respectively). Eighty-one percent reported travel distances ≥100 km and 27% reported travel distances ≥250 km. Among adults who likely required this specialist medical care, travel distance to specialist medical care was not associated with the proportion of adults reporting OSA symptoms in the absence of a sleep apnea diagnosis. Younger age, female sex, normal BMI, “just enough” or “some” money left over at the end of the month, and lower to moderate frequency of heavy alcohol consumption were also associated with higher proportions of adults reporting OSA symptoms in the absence of a sleep apnea diagnosis.

Sample characteristics by each distance quartile are shown in Supplemental Table 1 (Appendix A). No significant trends were observed.

Among adults who likely required this specialist medical care, the proportion reporting a sleep apnea diagnosis was low and unaffected by reported travel distance to specialist medical care (RR = 1.06; 95% CI: 0.79, 1.42; Table 5). We found marginal evidence of effect modification by sex at the 5% level of significance (*P*=0.05). In the cross-sectional data, there was some evidence of a significant relationship between travel distance to specialist medical care and reports of a sleep apnea diagnosis among men who likely required this specialist medical care (*P*=0.04); however, this relationship was only borderline significant at the 5% level of significance and followed no clear or interpretable trend, and the analysis of follow-up survey data showed no evidence of such a relationship (*P*=0.37). Sex was not found to be a meaningful effect modifier and was controlled for as a potential confounder. After adjustment for confounding variables, the association between travel distance to specialist medical care and sleep apnea diagnosis remained statistically nonsignificant (RR = 1.09; 95% CI: 0.82, 1.46; Table 5). There was no trend between the proportion of adults reporting a sleep apnea diagnosis and travel distance to specialist medical care (Figure 2).

#### 5.2.1. Analysis of Follow-Up Survey Data

Findings were consistent with our cross-sectional analysis. For adults who reported OSA symptoms in the absence of a sleep apnea diagnosis at baseline, travel distance to specialist medical care was not associated with reports of an incident sleep apnea diagnosis after five years (RR = 1.01; 95% CI: 0.98, 1.05; Table 6).

### 5.3. Sensitivity Analysis

A sensitivity analysis using multiple imputation revealed minimal impact of excluding participants with missing data for any of the study variables (data not shown).

## 6. Discussion

Using data from a large population-based rural health study conducted in Saskatchewan, we found that as travel distance to specialist medical care increased, so did the proportion of rural adult residents that reported OSA symptoms in the absence of a sleep apnea diagnosis. Among adults who likely required this specialist medical care and who would optimally be screened, diagnosed, and/or clinically managed appropriately, the proportion of sleep apnea diagnoses was low and unaffected by reported travel distance.

This study adds to a body of evidence that focuses on the population health impacts of poor access to specialist medical care in rural populations [47, 55, 56, 60–67], and specifically presents a novel analysis of the impact of travel distance to specialist medical care on sleep apnea diagnosis. Health care use is known to be lower in rural than in urban communities [60–63], and this has been attributed to increased travel distances and limited access to primary care and medical specialists [47, 55, 56, 64–67]. Health care access barriers are one possible risk factor for inadequate diagnosis and treatment of medical conditions [58, 70]. Even in the presence of accessible primary care, travel distance to specialist medical care is still a relevant concern for cases of sleep apnea because referral to a sleep medicine specialist for overnight, in-laboratory diagnostic testing, or at-home testing is required for diagnosis [71, 72]. Our finding of an increased proportion of rural adults reporting OSA symptoms in the absence of a sleep apnea diagnosis in association with increased travel distance to specialist medical care suggests decreased use of health care services by adults in remote rural communities.

Strengths and limitations of our analysis warrant comment. Our modeling strategy assessed for effect modification by biological sex, and then controlled for key confounding variables to reduce any differences that might arise for reasons other than travel distance to specialist medical care. Our analysis of follow-up survey data lent temporal evidence to our finding that the trend of an increasing proportion of adults reporting OSA symptoms in the absence of a sleep apnea diagnosis in association with increasing travel distance to specialist medical care was not attributable to differences in the frequency of diagnosis among adults who likely required this specialist medical care. Our findings suggest that once referred to a specialist, travel distance to specialist care is not associated with the sleep apnea diagnostic rate in rural populations. The low sleep apnea diagnostic rate may reflect other health care access barriers.

Self-reported travel distance may have resulted in exposure misclassification. Reporting a travel distance in kilometers may be difficult, especially if unfamiliar with where to get specialist care in Saskatchewan and if not having gone to see a specialist recently or ever. Participants may have over or underestimated travel distance as a result, which is supported by our comparison of reported travel distance and driving distance as determined by postal code, to the closest sleep center in the province. Although our exposure measure referred to all medical or surgical specialist services and was not specific to sleep specialist care, Saskatchewan's only two publicly funded sleep centers are located within the cities of Saskatoon and Regina [90, 91]. It is likely that most specialist medical and surgical services are provided out of these centers, especially given the general lack of medical specialists practicing in rural communities [47, 55]. Reliance on self-report data through a mailed questionnaire may have also led to underreporting the presence and/or degree of OSA symptoms due to social desirability bias and/or a lack of awareness that symptoms may be consistent with OSA. This potential outcome misclassification would have biased effect estimates for the first objective towards the null, assuming symptom underreporting was nondifferential across distance quartiles, and reduced the sample size and, therefore, power of the second objective. Though our analyses controlled for most known potential confounders, some degree of residual confounding is possible from measurement imprecision with self-report data.

Our sample was limited to adults living in rural Saskatchewan. Inclusion of adults from urban areas would have provided a useful basis of comparison. Our study could only evaluate the impact of travel distance to specialist medical care on OSA diagnosis between varying degrees of remoteness from such care. Our use of a sample that was exclusively rural in nature may have biased study findings in that effects that may be evident when you compare rural to urban populations would have been missed. Furthermore, in terms of external validity, the sample may not be representative of all rural populations, and the observed trend, rather than the magnitude of risk estimates, may only be generalized to rural communities without a strong metropolitan influence zone that have similar environmental and occupational exposures as Saskatchewan.

Our findings may have implications for health policy surrounding the diagnosis and treatment of OSA. In rural Saskatchewan, the largest reported travel distances to access specialist medical care (≥250 km) were associated with a greater proportion of adults reporting OSA symptoms in the absence of a sleep apnea diagnosis and suggest decreased use of health care services in remote populations. However, among adults who likely required this specialist medical care, the proportion of sleep apnea diagnoses was low and unaffected by reported travel distance. Factors other than travel distance may therefore be contributing to the low sleep apnea diagnostic rate. This remains important as undiagnosed and untreated OSA has serious implications on the health of people and populations [1, 10–24, 38–44], but effective treatments are available [25–37]. Health care access is an important determinant of health [58, 59] and barriers to the multiple dimensions of health care access (as per Levesque's model [70]) that have been established for other conditions (e.g., access to primary care, transportation barriers, and/or financial barriers) should be evaluated in future research that focuses on OSA. Interventional work on potential strategies to address and ameliorate any identified barriers is also warranted and results may provide important evidence to inform health care planning and delivery, which may be particularly impactful in rural and remote communities. Studies of interventional strategies, be their policies that foster better health care access through telemedicine [92–94] and portable diagnostic devices for in-home testing [4, 95–103], or innovative community outreach strategies that potentially increase access, especially among vulnerable and remote populations [104–106], are especially warranted.

## 7. Conclusions

Long travel distances may pose barriers to seeking health care in rural communities. In rural Saskatchewan, there was an increasing proportion of adults reporting OSA symptoms in the absence of a sleep apnea diagnosis in association with increasing travel distance to specialist medical care, which suggests decreased use of health care services in remote populations. However, among adults who likely required this specialist medical care, the proportion of sleep apnea diagnoses was low and unaffected by reported travel distance. Other health care access barriers may therefore be contributing to the low sleep apnea diagnostic rate. This remains important as undiagnosed and untreated OSA has serious implications on the health of people and populations, but effective treatments are available. Health care access barriers to the diagnosis and treatment of OSA require evaluation. Interventional work on potential strategies to address and ameliorate any identified barriers is warranted and results may provide important evidence to inform health care planning and delivery, which may be particularly impactful in rural and remote communities. Studies of interventional strategies, be their policies that foster better access through telemedicine and portable diagnostic devices for in-home testing, or innovative community outreach strategies that potentially increase access, especially among vulnerable and remote populations, are especially warranted.

## Figures and Tables

**Table 1:** Characteristics of the study sample (*n* = 6525; 3731 households).

Characteristics	Proportion (%)	Obstructive sleep apnea symptoms in the absence of a sleep apnea diagnosis (*n* = 2432; 37%)		*P* value^a^	Travel distance to specialist medical care ≥ 250 km (*n*=1651; 25%)		*P* value^a^
		*n*	Row %		*n*	Row %	
*Exposure*							
*Travel distance to specialist medical care (km)*							
<100	22	480	33.8	0.006	—	—	—
100–189	28	669	36.7		—	—	
190–249	25	622	38.1		—	—	
≥250	25	661	40.0		—	—	
*Potential confounders*							
*Age (years)* ^*b*^							
18–45	25	533	32.5	<0.001	393	23.9	0.08
46–55	26	695	41.6		439	26.3	
56–65	24	676	43.9		381	24.8	
>65	26	528	31.6		438	26.2	
*Sex*							
Female	50	922	28.5	<0.001	830	25.6	0.05
Male	50	1510	45.9		821	25.0	
*Body mass index (kg/m* ^*2*^)							
Normal (<25)	29	479	25.3	<0.001	463	24.5	0.38
Overweight (25–29.9)	41	1041	38.6		658	24.4	
Obese (≥30)	30	912	47.1		530	27.4	
*Education level*							
Postsecondary	41	947	35.2	0.005	668	24.9	0.02
Secondary or less	59	1485	38.7		983	25.6	
*Money left over at the end of the month*							
Some	60	1452	37.3	0.14	937	24.1	0.008
Just enough	21	489	35.4		328	23.7	
Not enough	19	491	39.3		386	30.9	
*Heavy alcohol consumption, more than 5 drinks on one occasion*							
Never	55	1217	34.0	<0.001	934	26.1	0.21
1/month or less	33	837	38.8		529	24.5	
1/week or less	10	320	48.1		161	24.2	
More than 1/week	2	58	47.2		27	22.0	
*Smoking status*							
Never	52	1118	32.7	<0.001	839	24.6	0.76
Past	36	981	42.3		596	25.7	
Current	12	333	42.2		216	27.4	

^a^ *P* value from Rao-Scott chi-square tests for significant difference in proportions with obstructive sleep apnea symptoms in the absence of a sleep apnea diagnosis/travel distance to specialist medical care ≥250 km between levels of each variable. ^b^Proportions add to 101% due to rounding.

**Table 2:** Results of multivariable log-binomial regression modeling likelihood of obstructive sleep apnea symptoms in the absence of a sleep apnea diagnosis in the study sample.

Variable	Unadjusted model RR (95% CI)	Adjusted model^a^ RR (95% CI)	*P* value
*Exposure*			
*Travel distance to specialist medical care (km)*			
<100	1.00 (referent)	1.00 (referent)	0.008
100–189	1.09 (0.99, 1.20)	1.08 (0.99, 1.19)	
190–249	1.13 (1.02, 1.25)	1.10 (1.00, 1.21)	
≥250	1.19 (1.08, 1.30)	1.17 (1.07, 1.29)	
*Potential confounders* ^*b*^			
*Age (years)*			
18–45	1.00 (referent)	1.00 (referent)	<0.001
46–55	1.28 (1.17, 1.40)	1.18 (1.08, 1.28)	
56–65	1.35 (1.23, 1.48)	1.20 (1.10, 1.32)	
>65	0.98 (0.88, 1.08)	0.91 (0.81, 1.01)	
*Sex*			
Females	1.00 (referent)	1.00 (referent)	<0.001
Males	1.63 (1.53, 1.73)	1.47 (1.37, 1.57)	
*Body mass index (kg/m* ^*2*^)			
Normal (<25)	1.00 (referent)	1.00 (referent)	<0.001
Overweight (25–29.9)	1.53 (1.39, 1.67)	1.35 (1.23, 1.48)	
Obese (≥30)	1.87 (1.71, 2.04)	1.59 (1.45, 1.74)	
*Money left over at the end of the month*			
Some	1.00 (referent)	1.00 (referent)	0.32
Just enough	0.95 (0.87, 1.03)	0.94 (0.87, 1.02)	
Not enough	1.05 (0.97, 1.15)	0.99 (0.91, 1.08)	
*Education level*			
Postsecondary	1.00 (referent)	1.00 (referent)	0.73
Secondary or less	1.11 (1.04, 1.18)	1.01 (0.95, 1.08)	
*Heavy alcohol consumption, more than 5 drinks on one occasion*			
Never	1.00 (referent)	1.00 (referent)	0.03
1/month or less	1.15 (1.07, 1.23)	1.00 (0.93, 1.07)	
1/week or less	1.44 (1.31, 1.58)	1.14 (1.04, 1.25)	
More than 1/week	1.40 (1.16, 1.68)	1.03 (0.86, 1.24)	
*Smoking status*			
Never	1.00 (referent)	1.00 (referent)	<0.001
Past	1.29 (1.20, 1.38)	1.16 (1.09, 1.25)	
Current	1.29 (1.17, 1.42)	1.20 (1.09, 1.32)	

^a^Model adjusted for age, sex, body mass index, money left over at the end of the month, education level, heavy alcohol consumption, and smoking status. Standard errors corresponding to confidence intervals were inflated to account for clustering of adults within households. ^b^Models adjusted for the other 6 potential confounders and travel distance to specialist medical care. Standard errors corresponding to confidence intervals were inflated to account for clustering of adults within households.

**Table 3:** Agreement between reported travel distance to specialist medical care and driving distance as determined by postal codes, to the closest sleep center in Saskatchewan.

Reported travel distance to specialist medical care (km)	Driving distance to the closest sleep center in Saskatchewan (km)			
	<100	100–189	190–249	≥250
<100	11%	16%	53%	20%
100–189	4%	36%	26%	34%
190–249	0%	11%	74%	15%
≥250	0%	3%	35%	62%

**Table 4:** Characteristics of adults who likely required this specialist medical care (*n* = 2813; 2230 households).

Characteristics	Proportion (%)	Obstructive sleep apnea symptoms in the absence of a sleep apnea diagnosis (*n* = 2432; 86%)		*P* value^a^	Travel distance to specialist medical care ≥ 250 km (*n* = 761; 27%)		*P* value^a^
		*n*	Row %		*n*	Row %	
*Exposure*							
*Travel distance to specialist medical care (km)*							
<100	19	480	87.6	0.71	—	—	—
100–189	28	669	85.4		—	—	
190–249	26	622	86.3		—	—	
≥250	27	661	86.9		—	—	
*Potential confounders*							
*Age (years)*							
18–45	21	533	91.1	0.003	140	23.9	0.12
46–55	29	695	85.3		224	27.5	
56–65	28	676	85.6		215	27.2	
>65	22	528	84.8		182	29.2	
*Sex*							
Females	36	922	89.9	<0.001	269	26.2	0.24
Males	64	1510	84.5		492	27.5	
*Body mass index (kg/m* ^*2*^)							
Normal (<25)	19	479	91.4	<0.001	128	24.4	0.52
Overweight (25–29.9)	41	1041	90.1		312	27.0	
Obese (≥30)	40	912	80.5		321	28.3	
*Education level*							
Postsecondary	39	947	86.3	0.80	287	26.1	0.40
Secondary or less	61	1485	86.6		474	27.6	
*Money left over at the end of the month*							
Some	59	1452	87.5	0.005	423	25.5	0.19
Just enough	20	489	87.8		153	27.5	
Not enough	21	491	82.4		185	31.0	
*Heavy alcohol consumption, more than 5 drinks on one occasion*							
Never	50	1217	86.2	0.03	402	28.5	0.26
1/month or less	34	837	87.6		248	25.9	
1/week or less	13	320	87.0		97	26.4	
More than 1/week	3	58	75.3		14	18.2	
*Smoking status*							
Never	46	1118	86.5	0.47	349	27.0	0.98
Past	41	981	85.8		314	27.5	
Current	13	333	88.3		98	26.0	

^a^ *P* value from Rao-Scott chi-square tests for significant difference in proportions with obstructive sleep apnea symptoms in the absence of a sleep apnea diagnosis/travel distance to specialist medical care ≥250 km between levels of each variable.

**Table 5:** Results of multivariable log-binomial regression modeling probability of a sleep apnea diagnosis among adults who likely required this specialist medical care.

Variable	Unadjusted model RR (95% CI)	Adjusted model^a^ RR (95% CI)	*P* value
*Exposure*			
*Travel distance to specialist medical care (km)*			
<100	1.00 (referent)	1.00 (referent)	0.32
100–189	1.17 (0.88, 1.57)	1.27 (0.95, 1.70)	
190–249	1.11 (0.83, 1.48)	1.21 (0.90, 1.62)	
≥250	1.06 (0.79, 1.42)	1.09 (0.82, 1.46)	
*Potential confounders* ^*b*^			
*Age (years)*			
18–45	1.00 (referent)	1.00 (referent)	<0.001
46–55	1.66 (1.22, 2.25)	1.71 (1.27, 2.32)	
56–65	1.63 (1.19, 2.22)	1.68 (1.23, 2.30)	
>65	1.71 (1.24, 2.36)	1.99 (1.41, 2.81)	
*Sex*			
Females	1.00 (referent)	1.00 (referent)	<0.001
Males	1.53 (1.24, 1.88)	1.58 (1.27, 1.96)	
*Body mass index (kg/m* ^*2*^)			
Normal (<25)	1.00 (referent)	1.00 (referent)	<0.001
Overweight (25–29.9)	1.16 (0.84, 1.60)	1.09 (0.79, 1.50)	
Obese (≥30)	2.28 (1.68, 3.08)	2.24 (1.65, 3.05)	
*Money left over at the end of the month*			
Some	1.00 (referent)	1.00 (referent)	0.02
Just enough	0.97 (0.75, 1.26)	1.01 (0.78, 1.30)	
Not enough	1.41 (1.13, 1.75)	1.39 (1.12, 1.73)	
*Education level*			
Postsecondary	1.00 (referent)	1.00 (referent)	0.05
Secondary or less	0.97 (0.80, 1.18)	0.82 (0.68, 1.00)	
*Heavy alcohol consumption, more than 5 drinks on one occasion*			
Never	1.00 (referent)	1.00 (referent)	0.12
1/month or less	0.90 (0.73, 1.012)	0.89 (0.72, 1.11)	
1/week or less	0.94 (0.70, 1.28)	0.93 (0.67, 1.28)	
More than 1/week	1.79 (1.19, 2.67)	1.65 (1.05, 2.58)	
*Smoking status*			
Never	1.00 (referent)	1.00 (referent)	0.60
Past	1.05 (0.86, 1.28)	0.91 (0.74, 1.11)	
Current	0.86 (0.63, 1.17)	0.89 (0.65, 1.22)	

^a^Model adjusted for age, sex, body mass index, money left over at the end of the month, education level, heavy alcohol consumption, and smoking status. Standard errors corresponding to confidence intervals were inflated to account for clustering of adults within households. ^b^Models adjusted for the other 6 potential confounders and travel distance to specialist medical care. Standard errors corresponding to confidence intervals were inflated to account for clustering of adults within households.

**Table 6:** Results of multivariable log-binomial regression modeling probability of an incident sleep apnea diagnosis after 5 years among adults with obstructive sleep apnea symptoms in the absence of a sleep apnea diagnosis (*n* = 1459; 1180 households).

Exposure	Proportion (%)	Obstructive sleep apnea symptoms in the absence of a sleep apnea diagnosis (row %)	Unadjusted model RR (95% CI)	Adjusted model^a^ RR (95% CI)	*P* value
*Travel distance to specialist medical care, km*					
<100	20	94.8	1.00 (referent)	1.00 (referent)	0.80
100–189	26	95.4	0.87 (0.44, 1.71)	1.00 (0.97, 1.04)	0.84^b^
190–249	26	96.4	0.69 (0.34, 1.41)	1.01 (0.98, 1.05)	
≥250	28	96.1	0.74 (0.37, 1.47)	1.01 (0.98, 1.05)	

^a^Model adjusted for age, sex, body mass index, money left over at the end of the month, education level, heavy alcohol consumption, and smoking status. Standard errors corresponding to confidence intervals were inflated to account for clustering of adults within households. ^b^ *P* value for trend test.

## Data Availability

Original microdata files for the Saskatchewan Rural Health Study used to support the findings of this study are available from Drs. Pahwa and Dosman at the Canadian Centre for Health and Safety in Agriculture, University of Saskatchewan, upon request.

## Supplementary Materials

Supplementary Materials
*Supplemental Table 1.* Characteristics of adults who likely required this specialist medical care (*n* = 2813; 2230 households) by distance quartile.
