# Supplementary material for: Distance to Specialist Medical Care and Diagnosis of Obstructive Sleep Apnea in Rural Saskatchewan
Source: Can Respir J. 2019 Jan 14;2019:1683124. doi: 10.1155/2019/1683124 (PMC6348862; doi:10.1155/2019/1683124)
Supplement: Supplementary Materials — Supplemental Table 1. Characteristics of adults who likely required this specialist medical care (n = 2813; 2230 households) by distance quartile. [file 1683124.f1.pdf]

Appendix A

Supplemental Table 1. Characteristics of Adults Who Likely Required This Specialist Medical Care (n = 2813; 2230 households) by distance quartile.

Characteristics	Travel distance to specialist medical care < 100km (n=548; 19%) Column %	Travel distance to specialist medical care 100-189km (n=783; 28%) Column %	Travel distance to specialist medical care 190-249km (n=721; 26%) Column %	Travel distance to specialist medical care ≥ 250km (n=761; 27%) Column %	*P* value[a]
**Age, years**					
18-45	19.9	23.6	20.9	18.4	.12
46-55	27.2	29.4	29.4	29.4	
56-65	32.1	24.0	29.3	28.3	
>65	20.8	23.0	20.4	23.9	
**Sex**					
Females	35.2	36.3	38.8	35.4	.24
Males	64.8	63.7	61.2	64.7	
**Body mass index, kg/m^2**					
Normal (<25)	18.8	18.4	20.7	16.8	.52
Overweight (25-29.9)	39.6	42.4	40.9	41.0	
Obese (≥30)	41.6	39.2	38.4	42.2	
**Education level**					
Postsecondary	37.4	39.1	41.6	37.7	.40
Secondary or less	62.6	60.9	58.4	62.3	
**Money left over at the end of the month**					
Some	58.9	60.0	61.6	55.6	.19
Just enough	18.4	21.6	18.6	20.1	
Not enough	22.6	18.4	19.8	24.3	
**Heavy alcohol consumption, more than 5 drinks on one occasion**					
Never	50.2	47.3	50.6	52.8	.26
1/month or less	33.8	34.7	34.8	32.6	
1/week or less	12.2	14.6	12.5	12.8	
More than 1/week	3.8	3.5	2.1	1.8	
**Smoking status**					
Never	46.2	46.0	45.9	45.9	.98
Past	41.1	39.6	40.8	41.3	
Current	12.8	14.4	13.3	12.9	

[a]*P* value from Rao-Scott chi-square tests for significant difference in proportions for travel distance to specialist medical care between levels of each variable.
